# Impact of internal heat source and rotation on a nonlocal thermoelastic solid with pores via the moore-gibson-thompson framework

**DOI:** 10.1038/s41598-026-58114-9

**Published:** 2026-06-22

**Authors:** Mohamed I. A. Othman, Samia M. Said, Ebtesam E. M. Eraki, Esraa M. Gamal

**Affiliations:** Department of Mathematics, Faculty of Science, Zagazig University, Zagazig, 44519 Egypt

**Keywords:** Moore-Gibson-Thompson theory, Rotation, Porous thermoelastic medium, Normal mode analysis, Internal heat source, Nonlocal parameter, Engineering, Materials science, Mathematics and computing

## Abstract

This study explores the combined effects of an internal heat source and rotation on a porous solid within the framework of nonlocal thermoelasticity as described by the Moore-Gibson-Thompson theory. By employing a non-dimensional approach, the governing equations were reformulated to highlight the underlying parameters and facilitate a more general analysis of the system. The resulting system of equations was subsequently solved using the normal mode method, yielding analytical expressions that describe the material’s displacement, temperature, and stress components. These solutions were further analyzed numerically using MATLAB, and graphical representations were generated to illustrate the effects of internal heating and rotation on the thermoelastic behavior of the porous medium. The findings reveal significant interactions between thermal and mechanical fields, offering deep insights into the performance and stability of advanced materials under complex loading conditions. This work not only advances the theoretical understanding of nonlocal thermoelasticity in porous solids but also provides practical implications for the design and optimization of engineering systems exposed to similar environmental influences.

## Introduction

Poro-thermoelasticity explores the interplay between thermal, mechanical, and fluid transport phenomena in porous materials, which are prevalent in geophysics, biomedical engineering, and energy systems. Marin ^1^ examined the significance and uniqueness of thermoelastic solids with voids, while Kumar and Rani ^2^ were the first to describe the distortions caused by dynamic loads in a thermoelastic solid with pores. Sharma and Kumar ^3^ extended this understanding by analyzing plane wave propagation in thermo-viscoelastic materials with pores. Deswal and Hooda ^4^ investigated a two-dimensional rotating magneto-thermoelastic half-space, incorporating voids and gravitational effects within the framework of two-temperature generalized thermoelasticity. Tomar et al. ^5^ explored plane waves in thermo-viscoelastic media, whereas Guo and Xiong ^6^ studied the impact of viscoelastic relaxation on poro-thermoelastic foundations. Gupta et al. ^7^ proposed a double poro-magneto-thermoelastic model that integrates micro-temperatures and memory-dependent heat transfer. Additionally, Othman et al. ^8^ analyzed the effects of gravity and viscosity on a fiber-reinforced thermoelastic solid with voids via the three-phase-lag model to capture their thermal and mechanical behavior. Many researchers have investigated various problems related to thermoelastic media with voids ^9–11^

Numerous studies have explored different phenomena in rotating media. For example, Schoenberg and Censor ^12^ investigated the propagation of plane harmonic waves in rationally elastic media without thermal effects, revealing that rotation introduces both concavity and anisotropy. Chand et al. ^13^ examined deformation, stress, and magnetic field distributions in uniformly rotating, homogeneous, isotropic elastic half-spaces that are thermally and electrically conductive. Similarly, Othman and Song ^14^ assessed the impact of rotation in magneto-thermoelastic materials, while Singh and Tomer ^15^ focused on how rotation affects the propagation of generalized thermoelastic plane waves. Additional research on the effects of rotation in various thermoelastic solids is presented by Abd-Alla et al. ^16^, Othman et al. ^17^, and Kalkal et al. ^18^.

Internal heat sources can profoundly influence a material’s thermoelastic behavior by introducing complex thermal gradients and stress distributions. These sources, originating from phenomena such as chemical reactions, electrical currents, friction, and nuclear processes, complicate the analysis of thermoelastic responses. For instance, Das and Kanoria ^19^ examined the challenges of magneto-thermoelastic interactions in functionally graded materials with periodically varying heat sources, while Sarkar and Lahiri ^20^ investigated the effects of a moving heat source at the material boundary, noting issues related to traction loss. In another study, Ailawalia and Singla ^21^ employed a dual phase lag model to capture the disturbances induced by a moving heat source. Abbas ^22^ applied an eigenvalue approach to solve problems in fractional-order magneto-thermoelastic materials exposed to a moving plane heat source, and Jain et al. ^23^ analyzed the response of fractional-order fiber-reinforced thermoelastic solids under similar conditions. Furthermore, Youssef and Al-Lehaibi ^24^ explored the behavior of a three-dimensional thermoelastic solid subjected to a moving heat source, while Zenkour et al. ^25^ investigated the coupled thermoelastic response of an infinite medium with a cylindrical hole under the influence of a moving heat source. Additional investigations by Das et al. ^26^ and Said ^27^ further underscore the complexities introduced by internal heat sources, including boundary heat flux effects and the influence of gravitational and.

mechanical forces on nonlocal thermoelastic solids.

Despite the significant progress achieved in generalized thermoelasticity, the combined effects of nonlocal elasticity, porosity, internal heat generation, and rotational fields within the Moore–Gibson–Thompson (MGT) thermoelastic framework remain largely unexplored. Most existing investigations have focused on either nonlocal thermo-elastic media, porous materials, or MGT heat conduction separately. The present study aims to bridge this gap by developing a unified mathematical model that accounts simultaneously for these important physical mechanisms. The novelty of this work lies in examining how internal heat sources and rotation influence the thermoelastic response of a nonlocal porous solid under the MGT heat transport theory. This comprehensive treatment provides new insights into wave propagation and field distributions that cannot be obtained from previously reported models. Further studies with the MGT theory can be found in ^28–34^.

Recent studies have examined various aspects of generalized thermoelasticity, including nonlocal effects, porous media, rotational fields, and advanced heat conduction theories. However, most of these investigations focus on a limited subset of these physical mechanisms. For example, some studies considered nonlocal thermoelastic responses without accounting for porosity or internal heat generation, while others investigated porous thermoelastic media under generalized heat conduction theories without incorporating rotational effects. In contrast, the present work integrates all these factors within the MGT framework, enabling a more comprehensive analysis of thermoelastic behavior. This distinction provides additional insight into the coupled influence of non-locality, porosity, rotation, and internal heat sources on the dynamic response of thermo-elastic solids.

In this work, we investigate the influence of an internal heat source and rotation on a.

porous solid within the scope of nonlocal thermoelasticity, employing the (MGT) theory.

Unlike classical thermoelastic models, which often fail to capture wave dispersion and non-Fourier effects, the MGT approach allows for a more comprehensive analysis of thermal and mechanical interactions in porous media. The inclusion of nonlocal effects further enhances the model’s applicability by accounting for micro-structural influences and long-range interactions, which are essential in modern engineering applications. Through analytical and numerical exploration, this study aims to elucidate the coupled behavior of heat transfer, mechanical deformation, and porosity dynamics in rotating, thermally loaded solids. The findings are expected to contribute to the advancement of nonlocal thermoelastic theories and their application in fields such as aerospace engineering, geomechanics, and energy materials.

### Formulation of the problem and basic equations

**Fig. 1:**
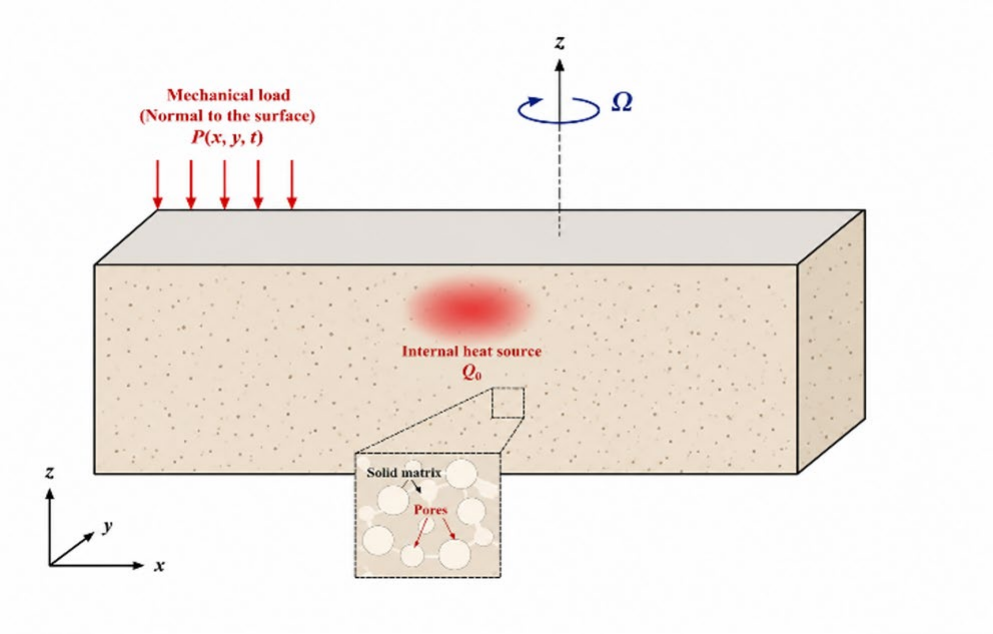
The schematic configuration of the problem.

To formulate the governing equations, the following assumptions are adopted:

(i) The material is homogeneous and isotropic; (ii) The thermoelastic response is linear; (iii) Deformations and temperature variations are sufficiently small so that higher-order nonlinear terms can be neglected; (iv) The nonlocal elasticity theory is applicable to account for size-dependent effects; (v) The medium contains distributed pores characterized by the adopted porosity model; (vi) The body rotates with a constant angular velocity $\Omega = \Omega \hat{n},$ where $\hat{\user2{n}}$ is a unit vector indicating the axis of rotation, $\Omega = (0,0,\Omega );$(vii) Heat conduction is governed by the Moore–Gibson–Thompson theory. Under these assumptions, the coupled thermoelastic field equations are derived and subsequently analyzed; and (viii) The problem is set in a two-dimensional context, where the deformation is confined to the $xy -$ plane, as Fig. 1, with the displacement vector given by ${\boldsymbol{u}} \equiv (u,v,0).$

The constitutive equations like Othman et al. ^8^ and Eringen ^35^

1 $$(1 - \varepsilon^{2} \nabla^{2} )\sigma_{ij} = \lambda e_{kk} \delta_{ij} + 2\mu e_{ij} - \;\gamma \theta \delta_{ij} + b\psi \delta_{ij} ,$$

2 $$e_{ij} = \frac{1}{2}(u_{i,j} + u_{j,i} ),e_{kk} = u_{,x} + v_{,y} .$$

The equations of motion can be written as ^12^:

3 $$\sigma_{ji,j} = \rho [u_{i,tt} + ({\boldsymbol{\varOmega}}\times ({\boldsymbol{\varOmega}}\, \times {\boldsymbol{u}}))_{i} + 2(\,{\boldsymbol{\varOmega}}\, \times {\boldsymbol{u}}_{,t} )_{i} ].$$

Heat conduction equation in the presence of the internal heat source

4 $$(K^{*} + K\frac{\partial }{\partial t})\nabla^{2} \theta = (1 + \tau_{0} \frac{\partial }{\partial t})[\rho C_{E} \theta_{,tt} + \gamma T_{0} \,e_{,tt} + \alpha_{3} T_{0} \psi_{,tt} - {\mathrm{Q}}_{,t} ]\,.$$

The equation of voids

5 $$\beta \psi_{,ii} - be - \alpha_{1} \psi - \alpha_{2} \psi_{,t} + \alpha_{3} \theta = \rho \alpha_{4} (1 - \varepsilon^{2} \nabla^{2} )\psi_{,tt} .$$

From Eq. (1) into Eq. (3), we get

6 $$B_{1} u_{,xx} + B_{2} v_{,xy} + B_{3} u_{,yy} - \gamma \theta_{,x} + b\psi_{,x} = \rho (1 - \varepsilon^{2} \nabla^{2} )[u_{,tt} - \Omega^{2} u + 2\Omega v_{,t} ],$$

7 $$B_{3} v_{,xx} + B_{2} u_{,xy} + B_{1} v_{,yy} - \gamma \theta_{,y} + b\psi_{,y} = \rho (1 - \varepsilon^{2} \nabla^{2} )[v_{,tt} - \Omega^{2} v + 2\Omega u_{,t} ],$$

where $$B_{1} = \lambda + 2\mu ,\,\,\,\,\,\,B_{2} = \lambda + \mu ,\,\,\,\,\,\,\,B_{3} = \mu .$$

The following dimensionless amounts are employed.

$$(x^{\prime},y^{\prime},\varepsilon^{\prime}) = \frac{{\omega_{1} }}{{c_{1} }}(x,y,\varepsilon ),\,\,(u^{\prime},v^{\prime})\, = \frac{{\rho c_{1} \omega_{1} }}{{\gamma T_{0} }}(u,v),\,\,\,\theta^{\prime} = \frac{\,1}{{T_{0} }}\theta ,\,\,\,\sigma_{ij}^{\prime } = \frac{1}{{\gamma T_{0} }}\sigma_{ij} ,\;\;\Omega^{\prime} = \frac{1}{{\omega_{1} }}\Omega ,$$

8 $$\begin{gathered} (t^{\prime},\tau_{0}^{\prime } ) = \omega_{1} (t,\tau_{0} ),Q^{\prime} = \frac{{c_{1}^{2} }}{{\omega_{1}^{2} T_{0} }}Q,\psi^{\prime} = \frac{{\rho c_{1}^{2} }}{{\gamma T_{0} }}\psi , \hfill \\ \omega_{1} = \frac{{\rho c_{1}^{2} C_{E} }}{{K^{*} }},c_{1}^{2} = \frac{\lambda + 2\mu }{\rho }. \hfill \\ \end{gathered}$$

The governing equations outlined previously take on the following format when employing the non-dimensional variables described in Eq. (8)

9 $$h_{1} u_{,xx} + h_{2} v_{,xy} + h_{3} u_{,yy} - \theta_{,x} + h_{4} \psi_{,x} = (1 - \varepsilon^{2} \nabla^{2} )[u_{,tt} - \Omega^{2} u - 2\Omega v_{,t} ],$$

10 $$h_{3} v_{,xx} + h_{2} u_{,xy} + h_{1} v_{,yy} - \theta_{,y} + h_{4} \psi_{,y} = (1 - \varepsilon^{2} \nabla^{2} )[v_{,tt} - \Omega^{2} v + 2\Omega u_{,t} ],$$

11 $$(d_{1} + d_{2} \frac{\partial }{\partial t})\nabla^{2} \theta = (1 + \tau_{0} \frac{\partial }{\partial t})[\theta_{,tt} + d_{3} \,e_{,tt} + d_{4} \psi_{,tt} - d_{5} {\mathrm{Q}}_{,t} ]\,,$$

12 $$\psi_{,ii} - a_{1} e - a_{2} \psi - a_{3} \psi_{,t} + a_{4} \theta = a_{5} (1 - \varepsilon^{2} \nabla^{2} )\psi_{,tt} .$$

where $$h_{\,1} = \frac{{B_{1} }}{{\rho c_{1}^{2} }},\,\,\,\,\,h_{\,2} = \frac{{B_{2} }}{{\rho c_{1}^{2} }},\,\,\,\,\,h_{\,3} = \frac{{B_{3} }}{{\rho c_{1}^{2} }},\,\,\,\,\,h_{\,4} = \frac{b}{{\rho c_{1}^{2} }},\,\,\,\,\,a_{1} = \frac{{bc_{1}^{2} }}{{\beta \omega_{1}^{2} }},\,\,\,\,\,a_{2} = \frac{{\alpha_{1} c_{1}^{2} }}{{\beta \omega_{1}^{2} }},$$ $$a_{3} = \frac{{\alpha_{2} c_{1}^{2} }}{{\beta \omega_{1} }},\,\,\,\,\,a_{4} = \frac{{\alpha_{3} \rho c_{1}^{4} }}{{\beta \gamma \omega_{1}^{2} }},\,\,\,\,\,a_{5} = \frac{{\alpha_{4} \rho c_{1}^{2} }}{\beta },\,\,\,\,\,\,d_{1} = \frac{{K^{*} }}{{\rho c_{1}^{2} C_{E} }},\,\,\,\,\,d_{2} = \frac{{K\omega_{1} }}{{\rho c_{1}^{2} C_{E} }},\,\,\,\,\,\,d_{3} = \frac{{\gamma^{2} T_{0} }}{{\rho^{2} c_{1}^{2} C_{E} }}\,,$$

$$d_{4} = \frac{{\alpha_{3} \,T_{0} \,\gamma }}{{\rho^{2} c_{1}^{2} C_{E} }},\,\,\,\,\,d_{5} = \frac{{\omega_{\,1} }}{{\rho c_{1}^{2} C_{E} }}.$$

### Normal mode analysis

Using normal mode analysis, the solution of the physical variable under study takes the following form

$$[u,v,\theta ,\sigma_{ij} ,\psi ](x,y,t) = [\overline{u},\overline{v},\overline{\theta },\overline{\sigma}_{ij} ,\overline{\psi }](x)\exp ({\mathrm{i}}ry - mt),$$

13 $$Q = \overline{Q}\exp (iry - mt),\overline{Q} = Q_{0} v_{0} ,$$

where $\overline{u}(x)$, etc. is the function amplitude of $u\,(x,y,\,t)$ etc.,

Using Eq. (13) in Eqs. (9)–(12), then we get

14 $$(A_{1} {\mathrm{D}}^{2} - A_{2} )\overline{u} + (A_{3} {\mathrm{D}}^{2} + {\mathrm{i}}rh_{2} {\mathrm{D}} - A_{4} )\overline{v} - {\mathrm{D}}\overline{\theta }{ + }h_{4} {\mathrm{D}}\overline{\psi } = 0,$$

15 $$(A_{3} {\mathrm{D}}^{2} - {\mathrm{i}}rh_{2} {\mathrm{D}} - A_{4} )\overline{u} + (A_{6} - A_{5} {\mathrm{D}}^{2} )\overline{v} + {\mathrm{i}}r\overline{\theta } - irh_{4} \overline{\psi } = 0,$$

16 $$a_{1} {\mathrm{D}}\overline{u} + {\mathrm{i}}ra_{1} \overline{v} - a_{4} \overline{\theta } + (A_{7} - A_{8} {\mathrm{D}}^{2} )\overline{\psi } = 0,$$

17 $$A_{9} {\mathrm{D}}\overline{u} + A_{10} \overline{v} + (A_{11} - A_{12} {\mathrm{D}}^{2} )\overline{\theta } + A_{13} \overline{\psi } = A_{14} {\mathrm{Q}}_{{0}} v_{0} ,$$

where $$A_{1} = h_{1} + \varepsilon^{2} (m^{2} - \Omega^{2} ),\,\,\,\,\,A_{2} = h_{3} r^{2} + (1 + \varepsilon^{2} r^{2} )(m^{2} - \Omega^{2} ),\,\,\,\,\,A_{3} = 2\Omega m\varepsilon^{2} ,$$

$$A_{4} = 2\Omega m(1 + \varepsilon^{2} r^{2} ),\,\,\,\,\,A_{5} = h_{3} + \varepsilon^{2} (m^{2} - \Omega^{2} ),\,\,\,\,\,A_{6} = h_{1} r^{2} + (1 + \varepsilon^{2} r^{2} )(m^{2} - \Omega^{2} ),$$

$$A_{7} = r^{2} + a_{2} - a_{3} m + a_{5} m^{2} ,\,\,\,A_{8} = 1 + a_{5} \varepsilon^{2} m^{2} ,\,\,\,A_{9} = d_{3} m^{2} (1 - \tau_{0} m),\,\,\,A_{10} = {\mathrm{i}}rd_{3} m^{2} (1 - \tau_{0} m),$$

$$A_{11} = m^{2} (1 - \tau_{0} m) + r^{2} A_{12} ,A_{12} = (d_{1} - d_{2} m),A_{13} = d_{4} m^{2} (1 - \tau_{0} m),A_{14} = - d_{5} m(1 - \tau_{0} m).$$

The resolution of Eqs. (14) - (17) yields the following results:

18 $$[{\mathrm{D}}^{8} - E_{1} {\mathrm{D}}^{6} + E_{2} {\mathrm{D}}^{4} - E_{3} D^{2} + E_{4} ]\,\overline{u}(x) = \frac{{A_{14} A_{15} {\mathrm{Q}}_{{0}} v_{0} }}{{A_{8} A_{12} A_{19} }},$$

where $$E_{1} = \frac{ - 1}{{A_{8} A_{12} (A_{3}^{2} + A_{1} A_{5} )}}[a_{1} h_{4} A_{5} A_{12} - A_{3}^{2} (A_{7} A_{12} + A_{8} A_{11} ) - A_{1} A_{5} (A_{7} A_{12} + A_{8} A_{11}$$

$$- A_{8} A_{12} (A_{1} A_{6} + A_{2} A_{5} + 2A_{3} A_{4} - h_{2}^{2} r^{2} ) - A_{5} A_{8} A_{9} ],$$

$$E_{2} = \frac{1}{{A_{8} A_{12} (A_{3}^{2} + A_{1} A_{5} )}}[A_{9} (A_{5} A_{7} + A_{6} A_{8} ) - a_{1} A_{5} A_{13} + A_{3}^{2} A_{7} A_{11} + A_{4}^{2} A_{8} A_{12} + a_{4} A_{3}^{2} A_{13}$$

$$+ A_{1} A_{5} A_{7} A_{11} + A_{1} A_{6} A_{7} A_{12} + A_{1} A_{6} A_{8} A_{11} + A_{2} A_{5} A_{7} A_{12} + A_{2} A_{5} A_{8} A_{11} + A_{2} A_{6} A_{8} A_{12}$$

$$+ \;2A_{3} A_{4} (A_{7} A_{12} + A_{8} A_{11} ) + a_{4} A_{1} A_{5} A_{13} - a_{1} h_{4} A_{5} A_{11} - a_{4} h_{4} A_{5} A_{9} - a_{1} h_{4} A_{6} A_{12} + {\mathrm{i}}rh_{2} A_{8} A_{10}$$

$$- \;r^{2} h_{2}^{2} (A_{7} A_{12} + A_{8} A_{11} ) - h_{2} r^{2} A_{8} A_{9} - {\mathrm{i}}rA_{1} A_{8} A_{10} - a_{1} h_{4} r^{2} A_{1} A_{12} + 2a_{1} h_{2} h_{4} r^{2} A_{12} ]$$,

$$E_{3} = \frac{ - 1}{{A_{8} A_{12} (A_{3}^{2} + A_{1} A_{5} )}}[a_{1} A_{6} A_{13} - A_{6} A_{7} A_{9} - A_{4}^{2} (A_{7} A_{12} + A_{8} A_{11} ) - A_{7} A_{11} (A_{1} A_{6} + A_{2} A_{5} )$$

$$- \;2A_{3} A_{4} A_{7} A_{11} - A_{2} A_{6} (A_{7} A_{12} + A_{8} A_{11} ) - a_{4} A_{13} (A_{1} A_{6} + A_{2} A_{5} + 2A_{3} A_{4} )\,\, + a_{1} h_{4} A_{6} A_{11}$$

$$+ a_{4} h_{4} A_{6} A_{9} - 2a_{1} h_{2} r^{2} A_{13} - {\mathrm{i}}rh_{2} A_{7} A_{10} + h_{2}^{2} r^{2} (A_{7} A_{11} + a_{4} A_{13} ) + a_{1} r^{2} A_{1} A_{13} + r^{2} h_{2} A_{7} A_{9}$$

$$+ \;{\mathrm{i}}rA_{10} (A_{1} A_{7} + A_{2} A_{8} ) + a_{1} h_{4} r^{2} (A_{1} A_{11} + A_{2} A_{12} - 2h_{2} A_{11} ) - a_{4} h_{2} h_{4} r^{2} A_{9} - {\mathrm{i}}ra_{4} h_{4} A_{10} (A_{1} - h_{2} )],$$

$$E_{4} = \frac{1}{{A_{8} A_{12} (A_{3}^{2} + A_{1} A_{5} )}}[A_{4}^{2} (A_{7} A_{11} + a_{4} A_{13} ) + A_{2} A_{6} (A_{7} A_{11} + a_{4} A_{13} ) - a_{1} r^{2} A_{2} A_{13} - {\mathrm{i}}rA_{2} A_{7} A_{10}$$

$$- \;a_{1} h_{4} r^{2} A_{2} A_{11} + {\mathrm{i}}ra_{4} h_{4} A_{2} A_{10} ].$$

Equation (18) can be decomposed as follows

19 $$({\mathrm{D}}^{{2}} - k_{1}^{2} )({\mathrm{D}}^{{2}} - k_{2}^{2} )\,\,({\mathrm{D}}^{{2}} - k_{3}^{2} )\,\,({\mathrm{D}}^{{2}} - k_{4}^{2} )\,\,\overline{u} \,(x) = \frac{{A_{14} A_{15} {\mathrm{Q}}_{{0}} v_{0} }}{{A_{8} A_{12} A_{19} }},$$

where $k_{n}^{2} \,\,(n = 1,\,2,\,3,\,4)$ are the roots of the equation:

20 $$k^{8} - E_{1} k^{6} + E_{2} k^{4} - E_{3} k^{2} + E_{4} = 0.$$

The delimited result of Eq. (18) is

21 $$\overline{u}(x) = \sum\limits_{i = 1}^{4} {N_{i} e^{{ - k_{i} x}} + \frac{{A_{14} A_{15} {\mathrm{Q}}_{{0}} v_{0} }}{{A_{8} A_{12} A_{19} E_{4} }}} .$$

Similarly,

22 $$\overline{v}(x) = \sum\limits_{i = 1}^{4} {H_{1i} N_{i} e^{{ - k_{i} x}} + \frac{{A_{14} A_{16} {\mathrm{Q}}_{{0}} v_{0} }}{{A_{8} A_{12} A_{19} E_{4} }}} ,$$

23 $$\overline{\theta }(x) = \sum\limits_{i = 1}^{4} {H_{2i} N_{i} e^{{ - k_{i} x}} + \frac{{A_{14} A_{17} {\mathrm{Q}}_{{0}} v_{0} }}{{A_{8} A_{12} A_{19} E_{4} }}} ,$$

24 $$\overline{\psi }(x) = \sum\limits_{i = 1}^{4} {H_{3i} N_{i} e^{{ - k_{i} x}} + \frac{{A_{14} A_{18} {\mathrm{Q}}_{{0}} v_{0} }}{{A_{8} A_{12} A_{19} E_{4} }}} .$$

Using the above equations, we get

25 $$\overline{\sigma}_{xx} (x) = \sum\limits_{i = 1}^{4} {H_{4i} N_{i} e^{{ - k_{i} x}} + G_{1} } ,$$

26 $$\overline{\sigma}_{xy} (x) = \sum\limits_{i = 1}^{4} {H_{5i} N_{i} e^{{ - k_{i} x}} + G_{2} } ,$$

where $$H_{1i} = \frac{{{\mathrm{i}}r(A_{2} - A_{1} k_{i}^{2} ) + (A_{3} k_{i}^{3} + {\mathrm{i}}rh_{2} k_{i}^{2} - A_{4} k_{i} )}}{{ir(A_{3} k_{i}^{2} - irh_{2} k_{i} - A_{4} ) - (A_{6} k_{i} - A_{5} k_{i}^{3} )}},$$

$$H_{2i} = \frac{{[(A_{7} - A_{8} k_{i}^{2} )(A_{1} k_{i}^{2} - A_{2} ) - a_{1} h_{4} k_{i}^{2} ] + [(A_{7} - A_{8} k_{i}^{2} )(A_{3} k_{i}^{2} - irh_{2} k_{i} - A_{4} ) + ira_{1} h_{4} k_{i} ]H_{1i} }}{{a_{4} h_{4} k_{i} - (A_{7} - A_{8} k_{i}^{2} )k_{i} }},$$

$$H_{3i} = \frac{{a_{1} k_{i} - ira_{1} H_{1i} + a_{4} H_{2i} }}{{(A_{7} - A_{8} k_{i}^{2} )}},H_{4i} = \frac{1}{F}[ - h_{1} k_{i} + irh_{5} H_{1i} - H_{2i} + h_{4} H_{3i} ],$$

$$H_{5i} = \frac{{h_{3} ({\mathrm{i}}r - k_{i} H_{1i} )}}{{(1 - \varepsilon^{2} k_{i}^{2} + \varepsilon^{2} r^{2} )}},\,\,\,\,G_{1} = \frac{{A_{14} {\mathrm{Q}}_{{0}} v_{0} (irh_{5} h_{16} - A_{17} + h_{4} A_{18} )}}{F},\,\,\,\,\,G_{2} = \frac{{irh_{3} A_{14} A_{15} {\mathrm{Q}}_{{0}} v_{0} }}{F},\,$$

$$F = (1 + \varepsilon^{2} r^{2} )A_{8} A_{12} A_{19} E_{4} .$$

## Boundary conditions

To determine the unknown constants, boundary conditions are imposed at the surface.

of the half-space $(x = 0).$

i. The thermal boundary condition,

27 $$\theta = f_{1} .$$

prescribes a constant thermal excitation at the boundary, where $f_{1}$ denotes the magnitude of the applied thermal loading.

ii. The mechanical conditions,

28 $$\sigma_{xx} = - f_{2} ,\sigma_{xy} = 0,$$

indicate that the surface is subjected to a uniform normal compressive load of magnitude $f_{2}$ while remaining free of tangential shear stresses.

iii. Furthermore, the condition imposed on the volume fraction field

29 $$\psi = 0.$$

assumes that no porosity disturbance is applied at the boundary. These conditions collectively describe a thermally excited porous half-space subjected to normal mechanical loading and are used to determine the unknown constants appearing in the analytical solution.

Incorporating Eqs. (23) – (26) into Eqs. (27)-(29), we obtain

30 $$\sum\limits_{{i = {1}}}^{{4}} {H_{2i} N_{i} } = S_{1} ,\sum\limits_{{i = {1}}}^{{4}} {H_{3i} N_{i} } = S_{2} ,\sum\limits_{{i = {1}}}^{{4}} {H_{4i} N_{i} } = - f_{2} - G_{1} ,\sum\limits_{i = 1}^{4} {H_{5i} N_{i} } = - G_{2} ,$$

where $$S_{1} = f_{1} - \frac{{A_{14} A_{17} {\mathrm{Q}}_{{0}} v_{0} }}{{A_{8} A_{12} A_{19} E_{4} }},\,\,\,\,\,\,$$$$S_{2} = \frac{{A_{14} A_{18} {\mathrm{Q}}_{{0}} v_{0} }}{{A_{8} A_{12} A_{19} E_{4} }}.$$

Using the matrix, the solution of Eq. (30) is:

31 $$\left( {\begin{array}{*{20}c} {N_{1} } \\ {N_{2} } \\ {N_{3} } \\ {N_{4} } \\ \end{array} } \right) = \left( {\begin{array}{*{20}c} {H_{21} } & {H_{22} } & {H_{23} } & {H_{24} } \\ {H_{31} } & {H_{32} } & {H_{33} } & {H_{34} } \\ {H_{41} } & {H_{42} } & {H_{43} } & {H_{44} } \\ {H_{51} } & {H_{52} } & {H_{53} } & {H_{54} } \\ \end{array} } \right)^{ - 1} \left( {\begin{array}{*{20}c} {S_{1} } \\ {S_{2} } \\ { - f_{2} - G_{1} } \\ { - G_{2} } \\ \end{array} } \right)\,.$$

The internal heat source parameter characterizes the intensity of heat generation within the material volume. Physically, it may arise from several mechanisms, including chemical reactions, electrical current flow, electromagnetic radiation absorption, frictional heating, or other internal energy-generation processes. In thermoelastic materials, internally generated heat contributes directly to the temperature field and consequently influences the mechanical response through thermal expansion and thermoelastic coupling. Therefore, variations in the internal heat source parameter can significantly alter the distributions of temperature, displacement, stress, and strain. Understanding its effect is particularly important in applications involving high-temperature environments, energy-conversion systems, microelectronic devices, nuclear engineering, and advanced functional materials.

## Numerical results and discussion

To compare the results using the (MGT) theory and Green-Naghdi (G-N III) theory, we examine the numerical outcomes based on the given physical constants (Othman et al. ^36^).

$$\lambda = 3.9\,{\mathrm{x}}10^{10} \,{\mathrm{N}}\,{.}\,{\mathrm{m}}^{{ - {2}}} ,\,\,\,\mu = 7.78\,{\mathrm{x}}10^{10} \,{\mathrm{N}}\,{.}\,{\mathrm{m}}^{{ - {2}}} ,\,\,\,\,\rho = 8954\,{\mathrm{kg}}\,{.}\,{\mathrm{m}}^{{ - {3}}} ,\,\,\,\,\,r = 0.9,\,\,\,\,\tau_{0} = 0.2{\mathrm{s}},\,\,\,$$

$$m_{0} = - 1.15,\,\,\,\,\xi = 0.2,\,\,\,\,\,m = m_{0} + {\mathrm{i}}\,\xi ,\,\,\,\,\,T_{0} = 293\,{\mathrm{K}},\,\,\,\,\,f_{1} = 0.4,\,\,\,\,\,C_{E} = 383.1\,\,{\mathrm{J}}\,{\mathrm{.kg}}^{{ - {1}}} {.}\,{\mathrm{K}}^{{ - {1}}} \,,\,\,\,\,$$

$${\mathrm{Q}}_{0} = 10\,{\mathrm{K}}\,,\,\,\,f_{2} = 0.3,\,\,\,\alpha_{4} = 1.753\,{\mathrm{x}}10^{ - 8} \,{\mathrm{N}}\,{.}\,{\mathrm{m}}^{{ - {2}}} \,,\,\,\,\alpha_{t} = 1.78\,{\mathrm{x}}10^{ - 5} K^{ - 1} ,\,\,\,\,b = 1.6\,{\mathrm{x}}10^{10} \,{\mathrm{N}}{\mathrm{.m}}^{{ - {2}}} ,$$

$$K^{*} = 386{\mathrm{w}}\,{.}\,{\mathrm{m}}^{{ - {1}}} {.}\,{\mathrm{K}}^{{ - {1}}} {\mathrm{.s}}^{{ - 1}} {,}\,\,\,K = 386{\mathrm{w}}\,{.}\,{\mathrm{m}}^{{ - {1}}} {.}\,{\mathrm{K}}^{{ - {1}}} {,}\,\,\,\,\alpha_{1} = 1.47\,{\mathrm{x}}10^{10} {\mathrm{N}}{\mathrm{.m}}^{{ - {2}}} ,\,\,\,\,\alpha_{2} = 7.78\,{\mathrm{x}}10^{ - 10} {\mathrm{N}}{\mathrm{.m}}^{{ - {2}}} ,$$

$$\alpha_{3} = 2\,{\mathrm{x}}10^{10} {\mathrm{N}}{\mathrm{.m}}^{{ - {2}}} ,\,\,\,\alpha_{4} = 1.753{\mathrm{x}}10^{ - 8} {\mathrm{N}}{\mathrm{.m}}^{{ - {2}}} ,\,\,\,v_{0} = 10\,{\mathrm{m}}{\mathrm{.s}}^{ - 1} ,\,\,\,\beta = 2\,{\mathrm{x}}10^{2} {\mathrm{N}}{\mathrm{.m}}^{{ - {2}}} ,\,\,\,y = 0\,.2.$$

The computations were carried out for non-dimensional time $t = 0.2.$ The physical.

variables are shown in graphs 2–16. Figures 2–6 exhibit the vertical displacement $v,$ the stress components $\sigma_{xx} ,\,\sigma_{xy} ,$ thermodynamic temperature distributions $\theta ,$ and change in volume fraction field $\psi$ in the lack and existence of the rotation.

**Fig. 2:**
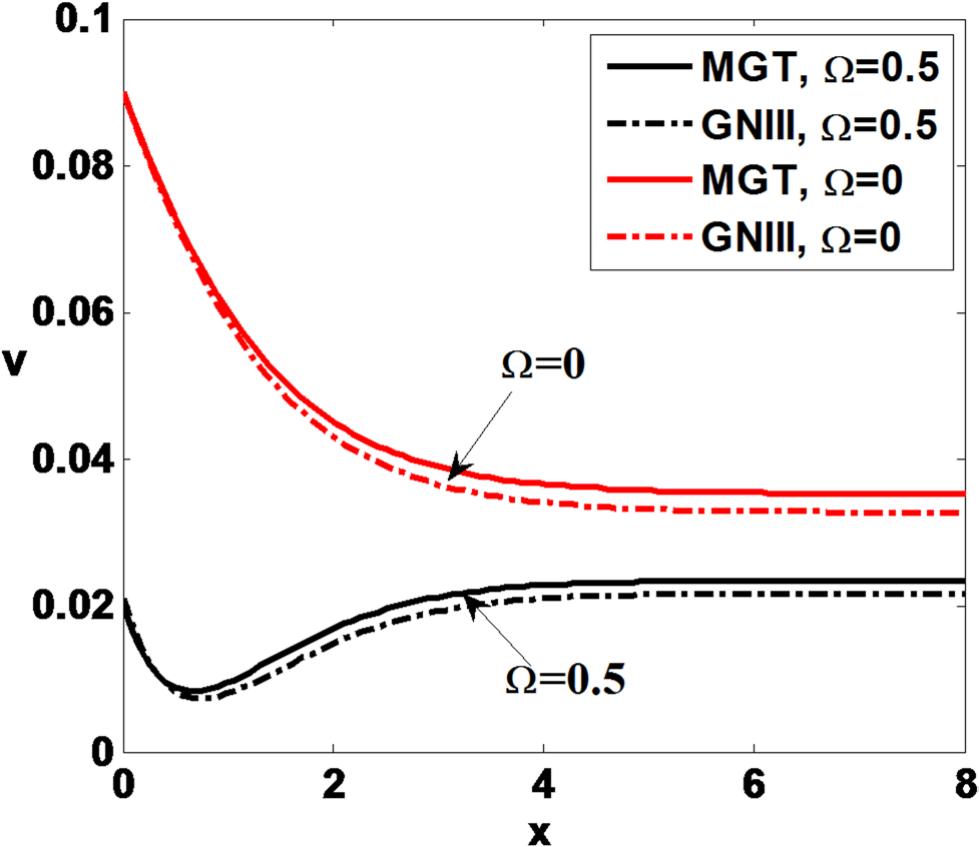
Variation of the vertical displacement $v$ with distance $x$ in the absence and presence of the rotation parameter $\Omega$ under the MGT and GNIII theories.

**Fig. 3:**
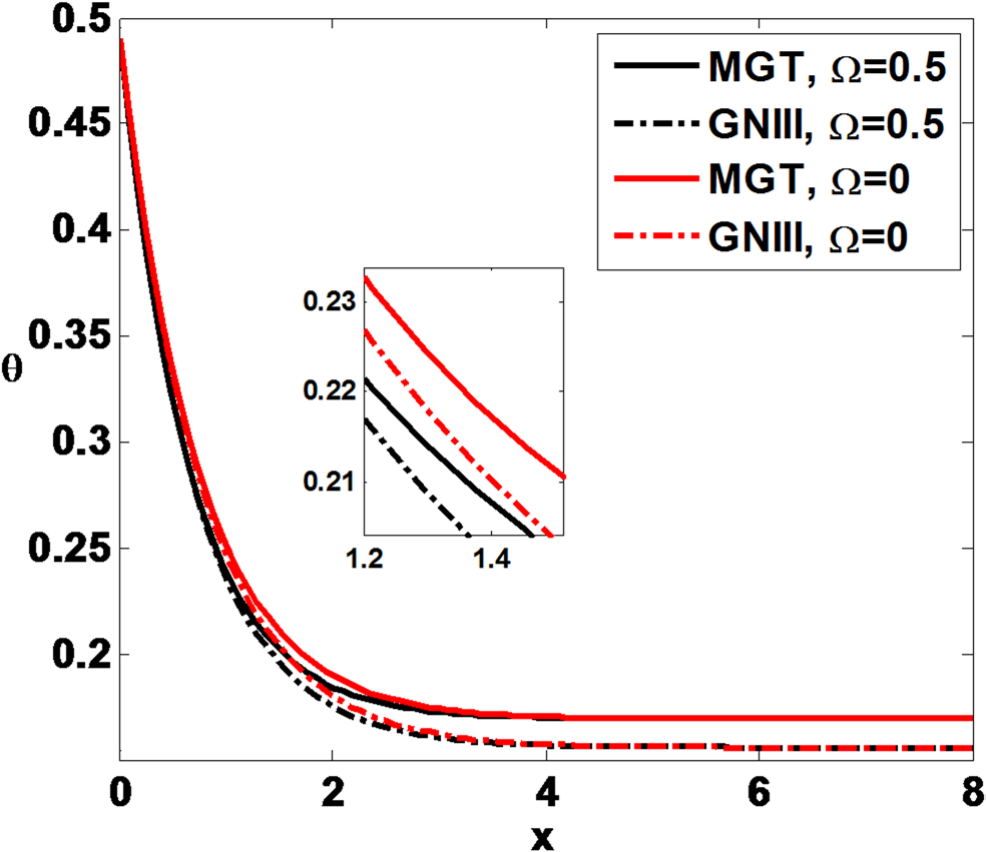
Variation of the Thermodynamics temperature $\theta$ with distance $x$ in the absence and presence of the rotation parameter $\Omega$ under the MGT and GNIII theories.

**Fig. 4:**
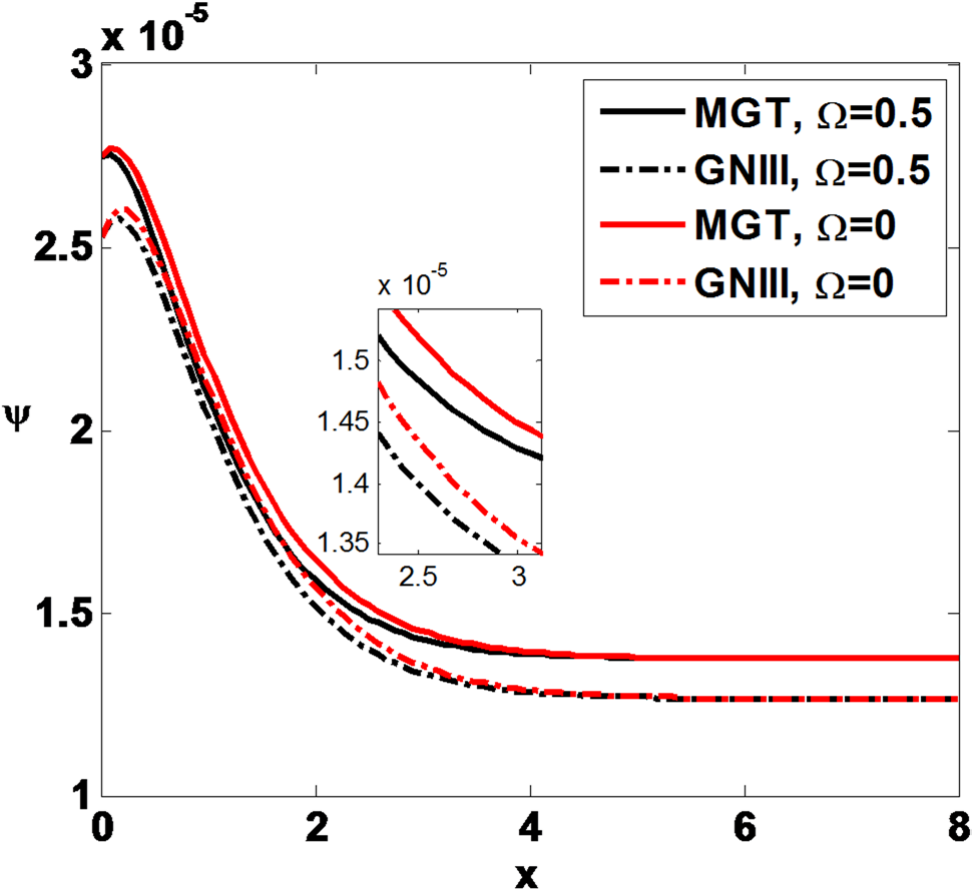
Variation of the volume fraction field $\psi$ with distance $x$ in the absence and presence of the rotation parameter $\Omega$ under the MGT and GNIII theories.

**Fig. 5:**
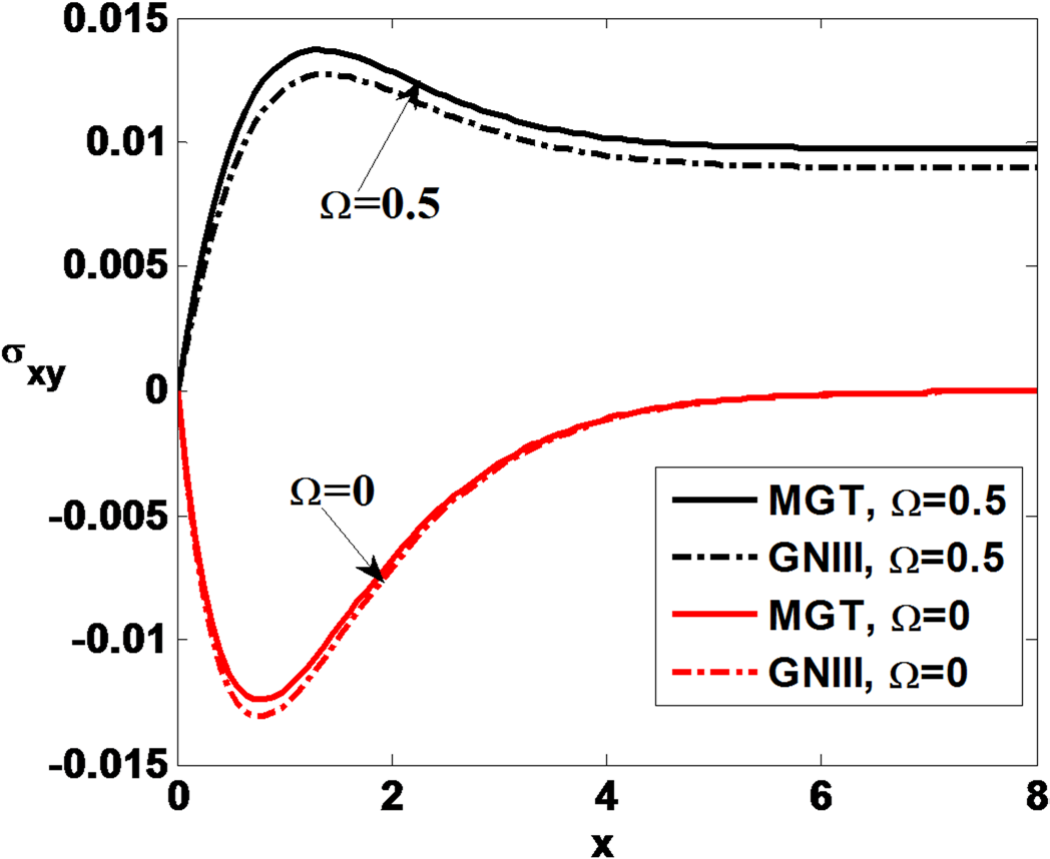
Variation of the stress component $\sigma_{xy}$ with distance $x$ in the absence and presence of the rotation parameter $\Omega$ under the MGT and GNIII theories.

**Fig. 6:**
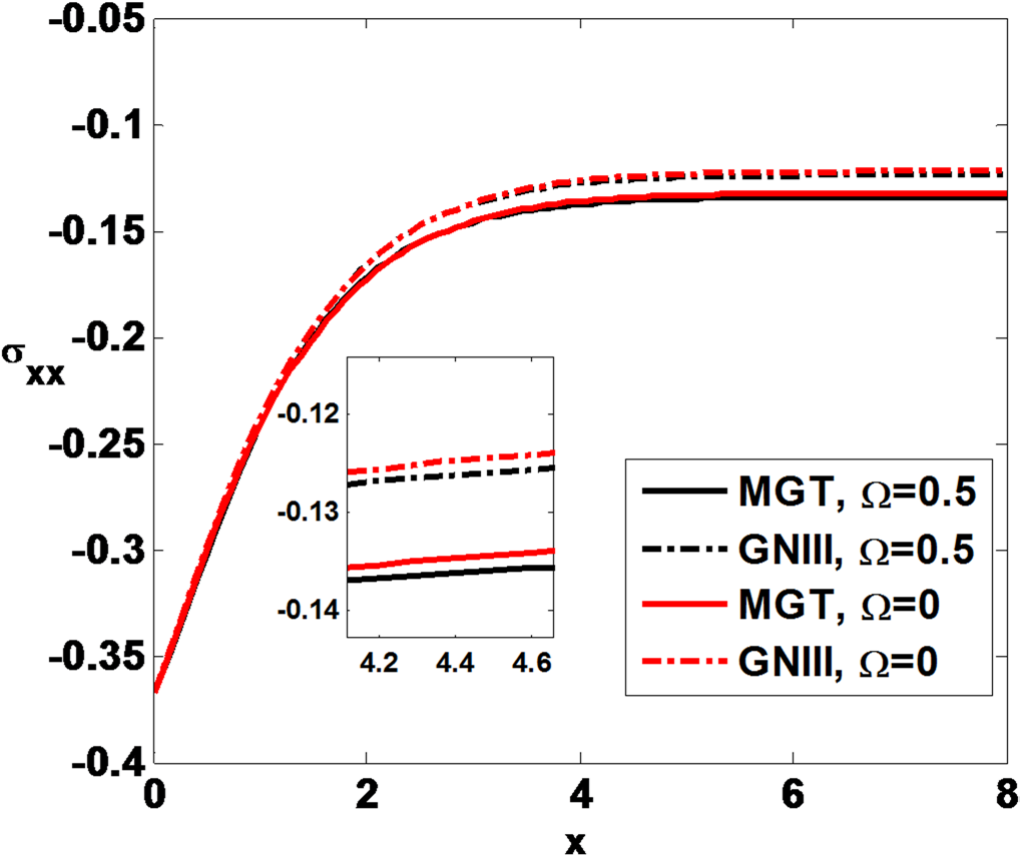
Variation of the stress component $\sigma_{xx}$ with distance $x$ in the absence and presence of the rotation parameter $\Omega$ under the MGT and GNIII theories.

Figure 2 illustrates the variation of the vertical displacement $v,$ under the influence of rotation. Near the boundary, the displacement initially attains positive values due to thermal expansion induced by the applied thermal loading. The generated temperature gradient causes the material particles to expand, producing an outward displacement in the vertical direction. As the rotational parameter increases, the magnitude of the vertical displacement decreases significantly. This behavior can be attributed to the additional inertial forces generated by rotation. In particular, the centrifugal force modifies the effective stress distribution within the medium, while the Coriolis force introduces a coupling between the particle motion and the angular velocity. These rotational effects oppose the thermally induced deformation and restrict the free expansion of the material. Consequently, part of the energy that would otherwise contribute to vertical motion is redistributed through rotational inertia, leading to a reduction in the displacement amplitude. Furthermore, rotation tends to increase the effective stiffness of the medium against deformation. As a result, the propagation of thermoelastic disturbances becomes more constrained, causing the displacement field to decay more rapidly with distance from the boundary. Therefore, the observed reduction in $v,$ demonstrates the stabilizing influence of rotation on the thermoelastic response of the porous nonlocal medium within the Moore–Gibson–Thompson framework. Figure 3 illustrates the spatial distribution of the thermal temperature field $\theta$ in a porous thermoelastic medium subjected to rotation, revealing insights into heat transport behavior under mechanical rotation**.** The temperature profile exhibits a characteristic exponential-like decay with distance, which is typical of diffusive heat conduction thermal energy flows away from the heat source, and the system tends toward equilibrium as thermal gradients diminish. This decay pattern reflects the dominant role of conduction mechanisms over convective or inertial effects in the medium. The porous structure allows for some thermal buffering or storage, but it is still governed by classical diffusion principles unless strongly coupled with fluid flow or intense internal heating. Notably, the comparison between the MGT and GNIII models shows only minor differences in the temperature distribution, implying that the rotational effects have limited impact on the thermal field. Figure 4 presents the evolution of the volume fraction field $\psi ,$ which characterizes micro-structural properties such as porosity, diffusion concentration, or void distribution within a porous thermoelastic material**.** The observed behavior a rapid initial decrease followed by stabilization reflects the material’s transient response to thermo-mechanical coupling under both the MGT and GNIII theories. Initially, the system undergoes a sharp change in $\psi ,$ indicating a rapid redistribution of mass or micro-structural volume, likely triggered by thermal expansion and stress-induced diffusion. Figure 5 illustrates the behavior of the shear stress component $\sigma_{xy}$ under the influence of rotational motion, highlighting how angular velocity affects the internal stress distribution in a thermoelastic medium**.** The comparison between the rotating $(\Omega = 0.5)$ and non-rotating $(\Omega = 0)$ cases reveals clear differences in the material’s mechanical response. In the absence of rotation $(\Omega = 0),$ the stress component $\sigma_{xy}$ initially decreases sharply, reaching a minimum due to the rapid mechanical adjustment to applied thermal and boundary conditions. This is followed by a gradual stabilization, indicating the system’s progression toward equilibrium as internal forces balance out. When rotation is introduced $(\Omega = 0.5),$ the shear stress profile changes noticeably. The curves shift upward, showing higher peak stress values and a smoother decay, indicating that rotation significantly amplifies the shear stress throughout the medium. Figure 6 presents the evolution of the normal stress component $\sigma_{xx} ,$ examining how rotation influences the axial stress response in a thermo-elastic medium**.** The results indicate that in both the presence and absence of rotation, $\sigma_{xx}$ increases with position or time, reflecting the system’s internal buildup of stress due to thermal expansion and mechanical coupling. While rotation ($(\Omega \ne 0\backslash \Omega = 0)$ introduces additional inertial forces such as Coriolis and centrifugal effects the overall trend of $\sigma_{xx}$ remains qualitatively unchanged compared to the non-rotating case. This suggests that, in this context, rotation has a minimal or indirect effect on the axial stress component**,** particularly if the geometry or loading is such that rotation primarily influences shear or transverse deformation modes rather than axial ones. Physically, this behavior implies that the primary driver of axial stress is thermal loading, with heat-induced expansion producing tensile or compressive stresses along the direction of wave propagation or boundary constraint. Since rotation acts more significantly on components perpendicular to the rotation axis (i.e., affecting shear or lateral interactions), its influence on $\sigma_{xx}$ is more subtle and may not be sufficient to alter the overall stress development pattern.

**Fig. 7:**
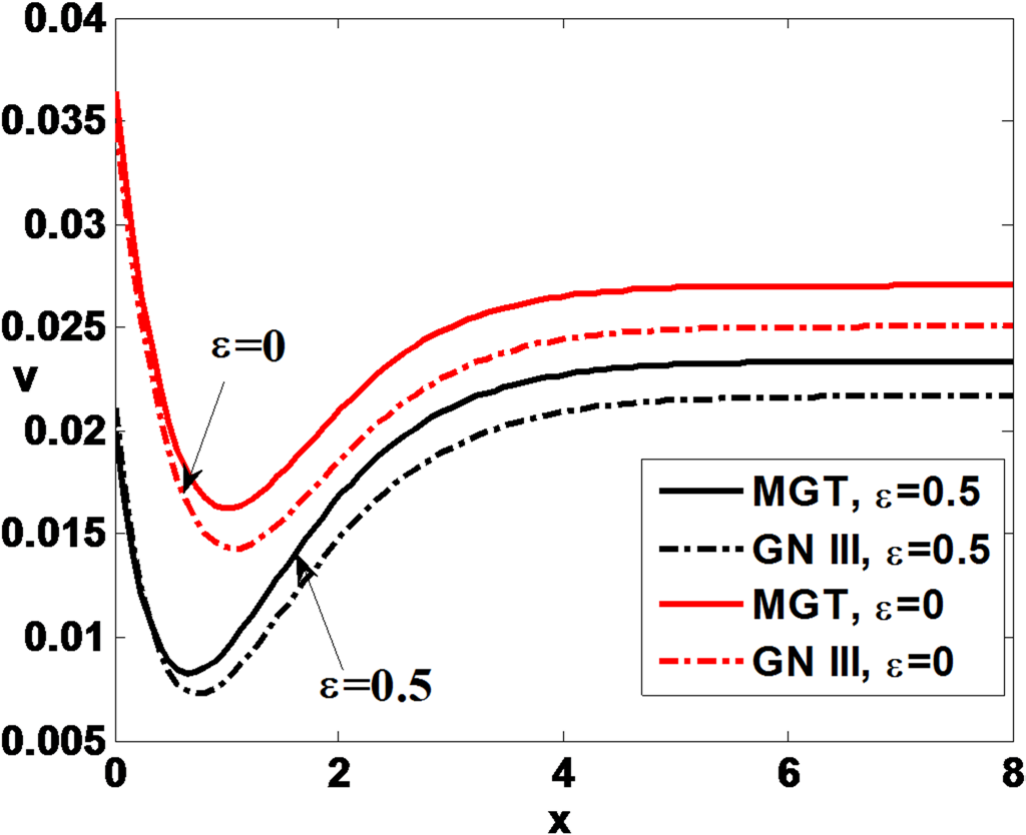
Variation of the vertical displacement $v$ with distance $x$ in the absence and presence of the nonlocal parameter $\varepsilon$ under the MGT and GNIII theories.

**Fig. 8:**
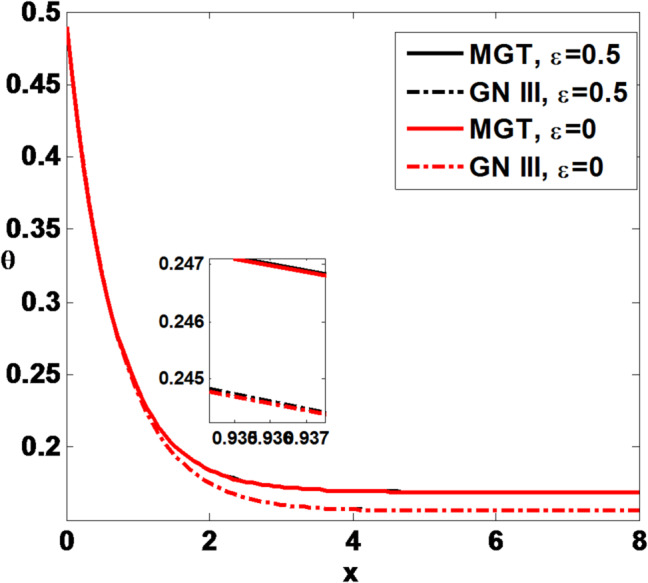
Variation of the thermal temperature $\theta$ with distance $x$ in the absence and presence of the nonlocal parameter $\varepsilon$ under the MGT and GNIII theories.

**Fig. 9:**
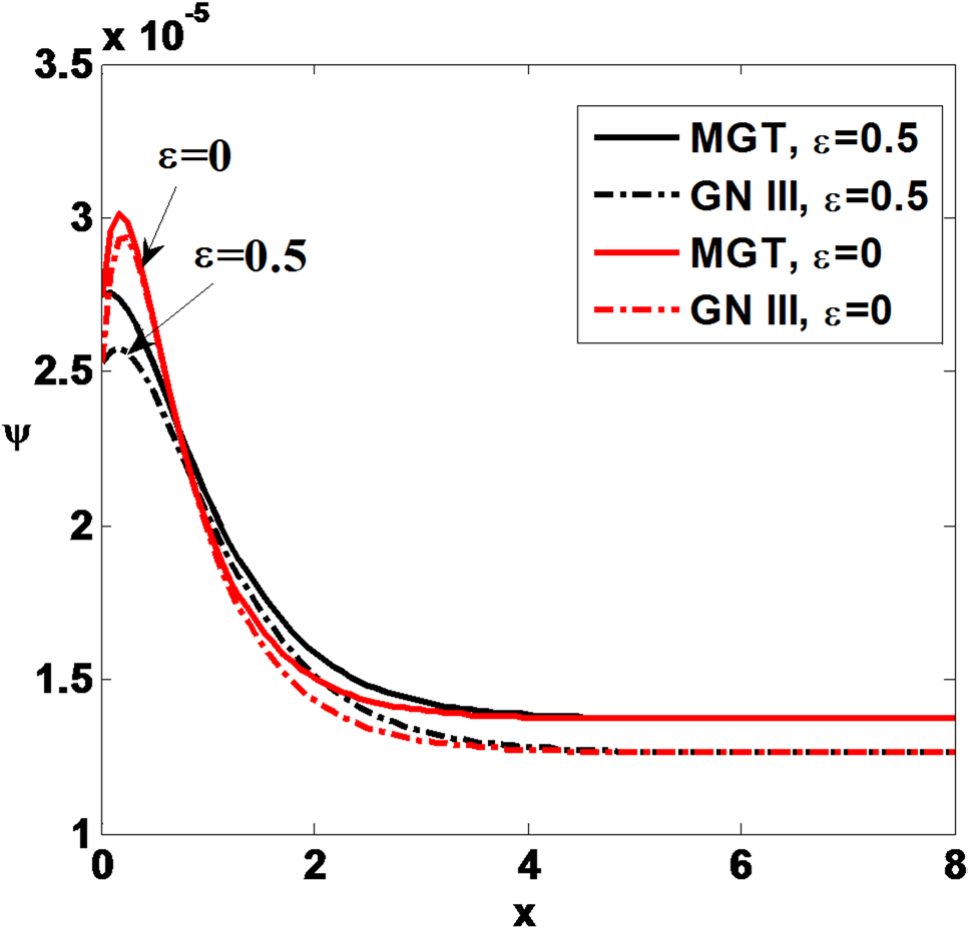
Variation of the volume fraction field $\psi$ with distance $x$ in the absence and presence of the nonlocal parameter $\varepsilon$ under the MGT and GNIII theories.

**Fig. 10:**
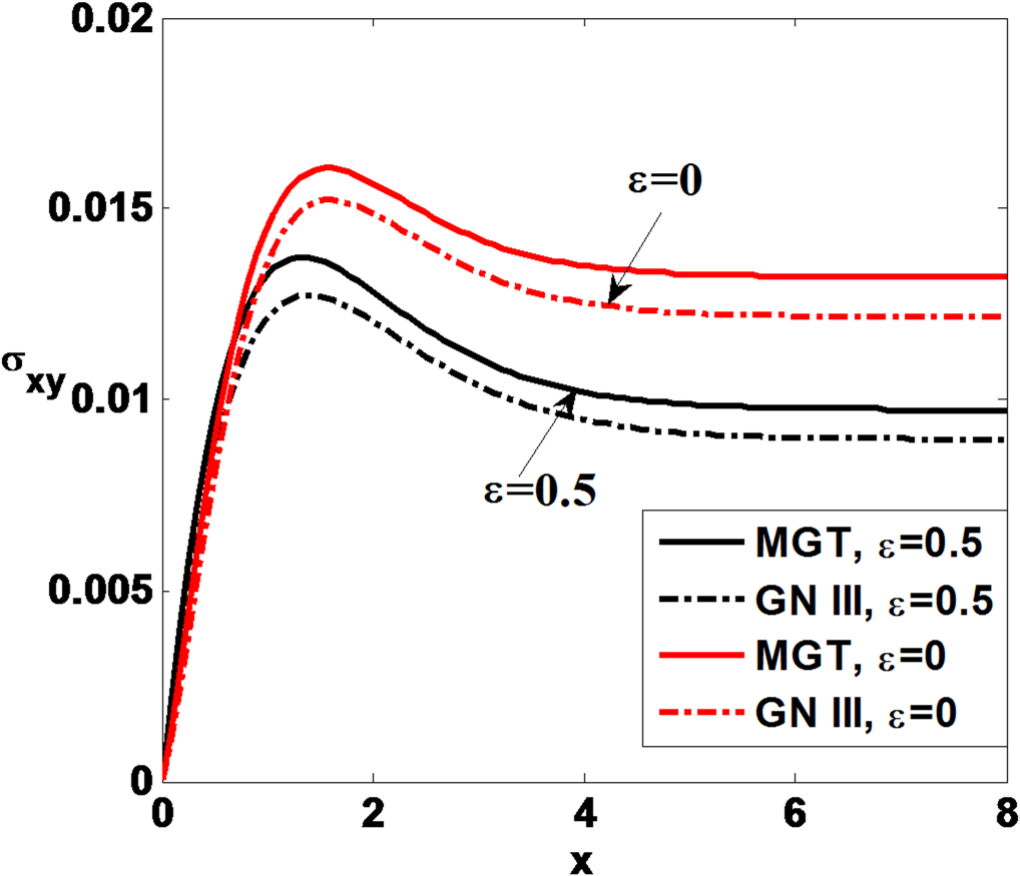
Variation of the stress component $\sigma_{xy}$ with distance $x$ in the absence and presence of the nonlocal parameter $\varepsilon$ under the MGT and GNIII theories.

**Fig. 11:**
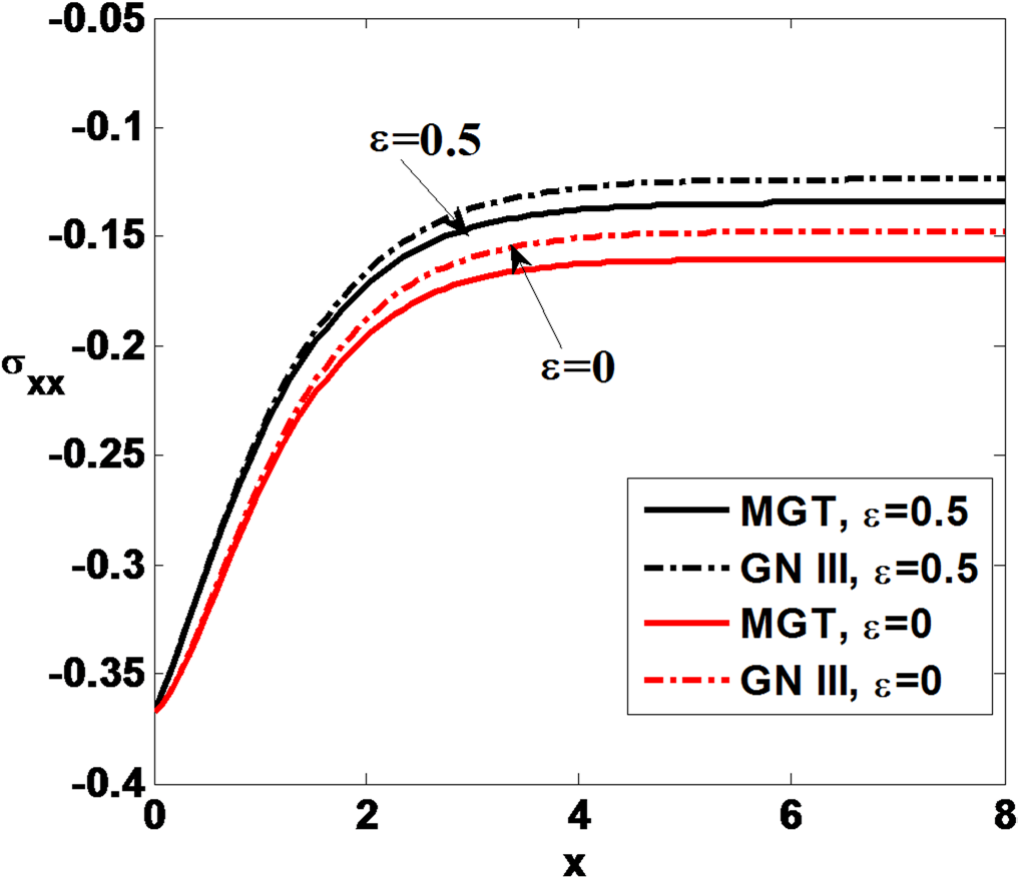
Variation of the stress component $\sigma_{xx}$ with distance $x$ in the absence and presence of the nonlocal parameter $\varepsilon$ under the MGT and GNIII theories.

Figures 7–11 exhibit the vertical displacement $v$, the stress components $\sigma_{xx} ,\,\sigma_{xy} ,$ thermo-dynamic temperature distributions $\theta ,$ and change in volume fraction field $\psi$ in the lack and existence of the locality. Figure 7 illustrates the behavior of the vertical displacement component $v,$ emphasizing how nonlocal elasticity and different thermo-elastic theories influence the deformation response of the medium**.** The figure clearly demonstrates that the inclusion of the nonlocal parameter $\varepsilon$ significantly alters the displacement profile, especially in the early region where thermal or mechanical disturbances are strongest. When $\varepsilon = 0$(local elasticity), both the MGT and GNIII theories predict higher displacement magnitudes, particularly in the initial region where wave-like or oscillatory behavior may appear due to rapid thermal expansion. This suggests that in the absence of nonlocal effects, the material responds more strongly to localized thermal or mechanical input, leading to sharper and more intense deformation. However, when $\varepsilon = 0.5,\,$ the displacement curves become smoother and less intense, indicating that the nonlocal parameter introduces a damping-like effect. Figure 8 presents the distribution of the thermal temperature field $\theta$ along the spatial domain $0 \le x \le 8,$ highlighting how heat dissipates within the medium under both local and nonlocal thermoelastic formulations. In both cases whether the nonlocal parameter is included or not $\theta$ decreases steadily with increasing $x,$ reflecting the natural decay of thermal energy away from the heat source or boundary. This behavior corresponds to the classical expectation of heat conduction: thermal energy diffuses from regions of high temperature toward cooler regions, driving a spatial decline in temperature. The smooth decrease in $\theta$ demonstrates that the system is attempting to reach thermal equilibrium as heat propagates through the medium. Figure 9 illustrates the evolution of the volume fraction field $\psi ,$ which characterizes micro-structural variations such as porosity or diffusion-driven concentration changes, under the influence of the nonlocal parameter $\varepsilon$ and different thermoelastic theories. For both MGT and GNIII models, the behavior of $\psi$ follows a similar trend: an initial rise to a peak value, followed by a gradual decay and stabilization as the system approaches equilibrium. This transient peak reflects a region of intense micro-structural activity, driven by rapid thermal or mechanical excitation such as heat-induced expansion or stress-assisted diffusion especially near boundaries or where gradients are steepest. When the nonlocal parameter is introduced $(\varepsilon = 0.5),\,$ the peak magnitude of $\psi$ is slightly reduced compared to the local case $(\varepsilon = 0).$ Figure 10 illustrates the distribution of the shear stress component $\sigma_{xy} ,$ highlighting the influence of the nonlocal parameter $\varepsilon$ and comparing the responses under the MGT and GNIII thermoelastic theories**.** The results show that the shear stress is higher when $\varepsilon = 0$ (i.e., under classical local elasticity) and decreases when $(\varepsilon = 0.5).\,$ This indicates that the incorporation of nonlocal effects reduces the magnitude of shear stress. Physically, the nonlocal parameter $\varepsilon$ accounts for the spatial interactions between material points beyond immediate neighbors, modeling the internal length scale effects of the microstructure. When $\varepsilon = 0,$ the stress at a point depends not only on the local strain but also on the strain in the surrounding region. Figure 11 illustrates the effect of the nonlocal parameter $\varepsilon$ on the distribution of the normal stress component $\sigma_{xx} ,$ providing insight into the role of small-scale interactions and material microstructure in thermoelastic stress response. In both the MGT and GNIII frameworks, it is observed that when $\varepsilon = 0.5,$ the values of $\sigma_{xx}$ are slightly higher compared to the case where $\varepsilon = 0.$ Physically, the nonlocal parameter $\varepsilon$ accounts for the influence of long-range intermolecular forces or micro-structural interactions in the material. When $\varepsilon > 0,$ the stress at a given point is not determined solely by local deformation, but also by the state of the surrounding material, effectively introducing a form of spatial coupling or internal material memory. As a result, when nonlocal effects are included $(\varepsilon = 0.5),$ the material resists deformation more strongly, and this resistance manifests as higher stress magnitudes.

**Fig. 12:**
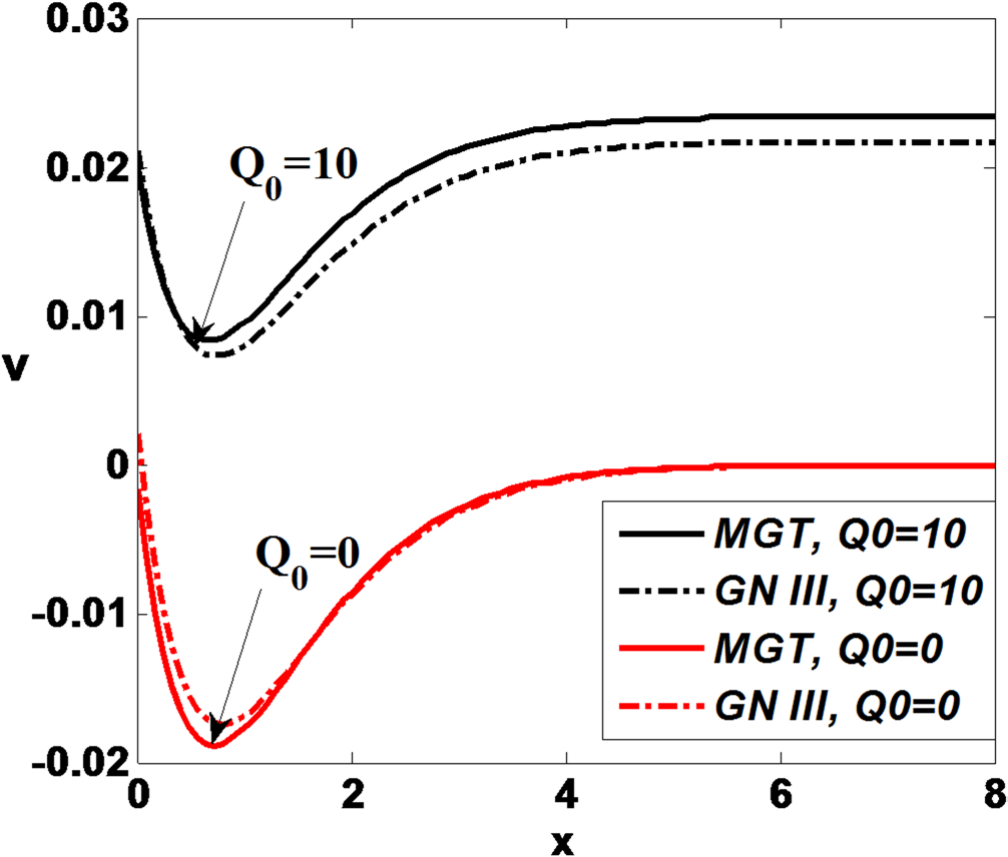
Variation of the vertical displacement $v$ with distance $x$ in the absence and presence of an internal heat source under the MGT and GNIII theories.

**Fig. 13:**
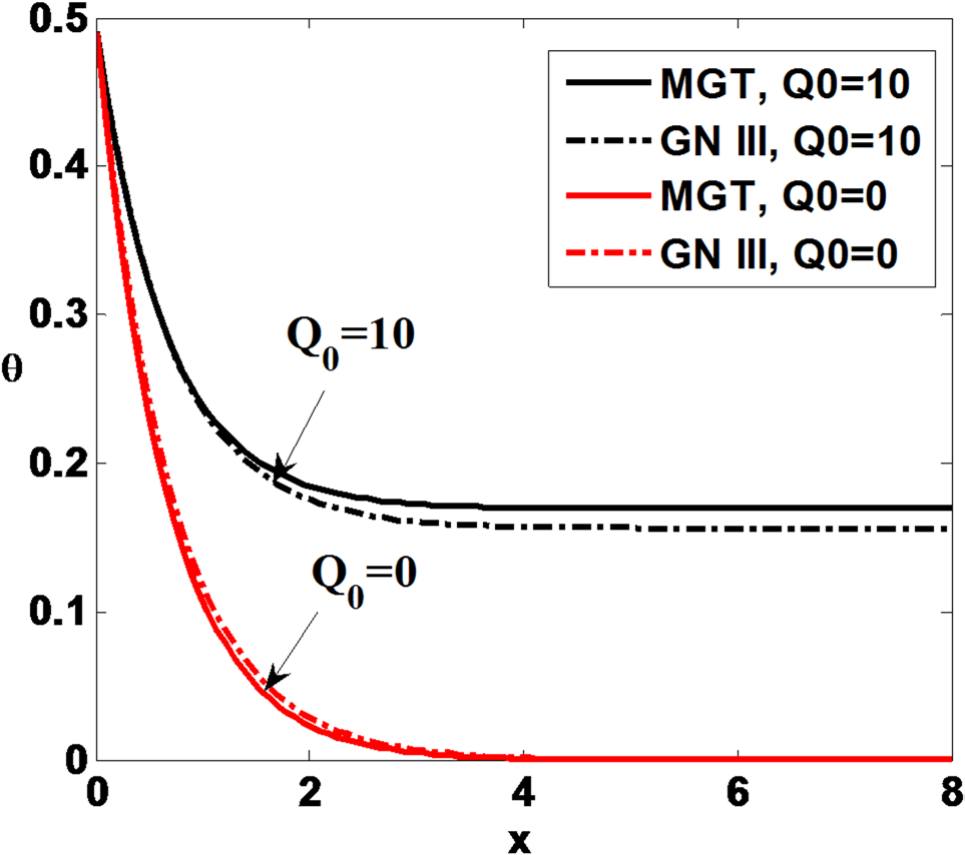
Variation of the thermal temperature $\theta$ with distance $x$ in the absence and presence of an internal heat source under the MGT and GNIII theories.

**Fig. 14:**
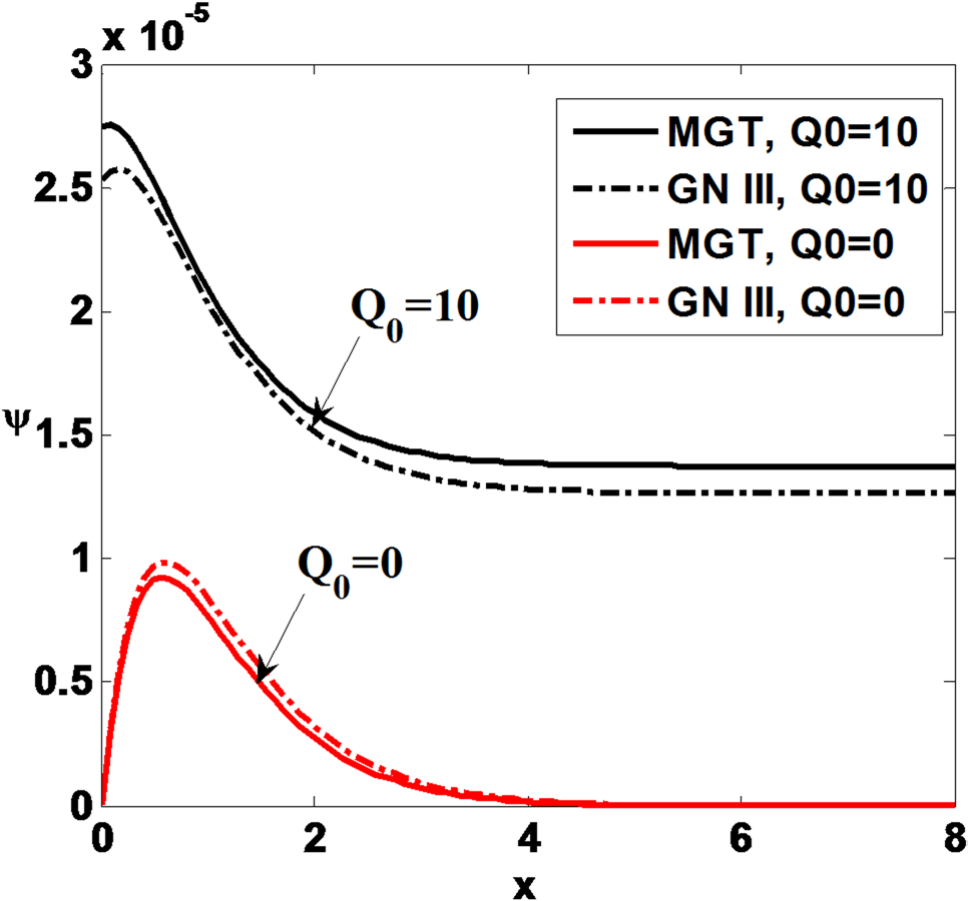
Variation of the volume fraction field $\psi$ with distance $x$ in the absence and presence of an internal heat source under the MGT and GNIII theories.

**Fig. 15:**
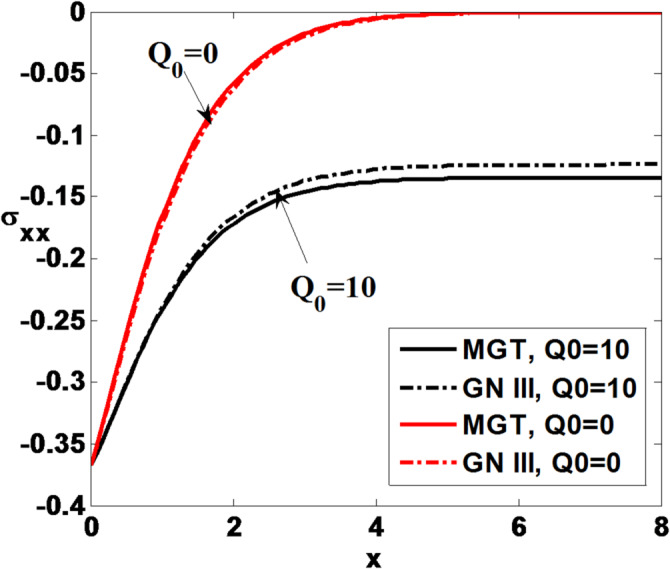
Variation of the stress component $\sigma_{xx}$ with distance $x$ in the absence and presence of an internal heat source under the MGT and GNIII theories.

**Fig. 16:**
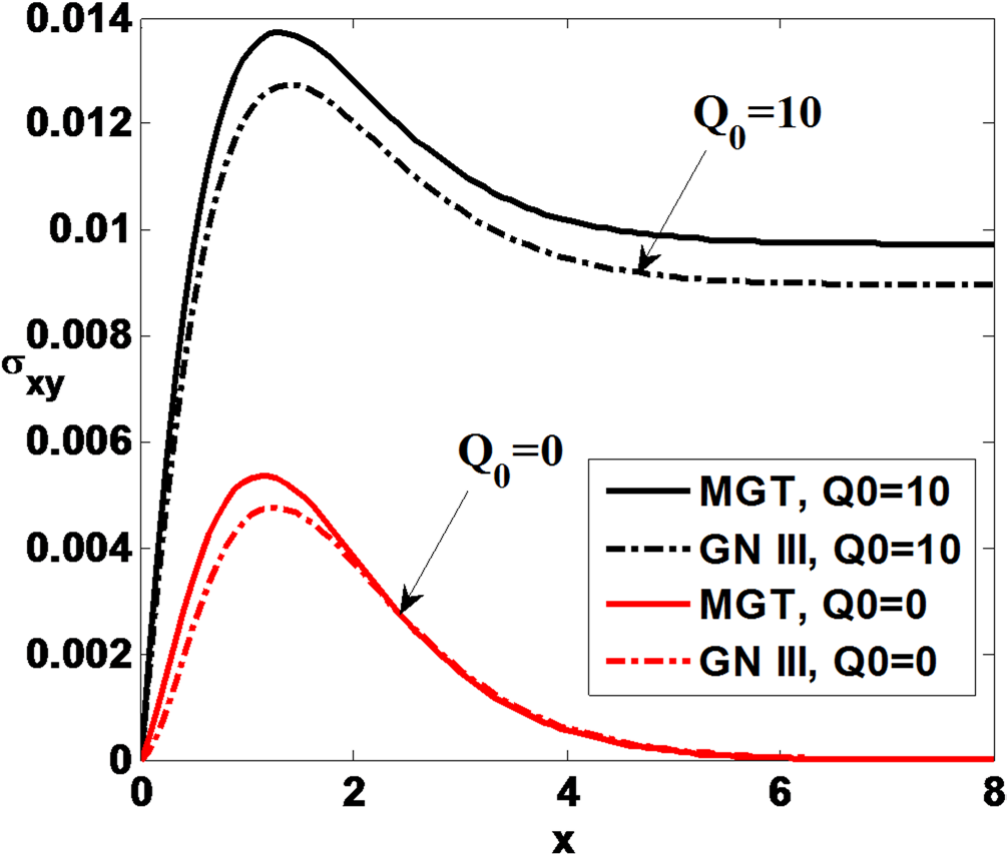
Variation of the stress component $\sigma_{xy}$ with distance $x$ in the absence and presence of an internal heat source under the MGT and GNIII theories.

Figures 12–16 exhibit the vertical displacement $$v,$$ the stress components $$\sigma_{xx} ,\,\sigma_{xy} ,$$ thermodynamic temperature distributions $$\theta ,$$ and change in volume fraction field $$\psi$$ in the deficiency and proximity of a moving internal heat source. Figure 12 illustrates the behavior of the vertical displacement component $$v$$ under the influence of an internal heat source $${\mathrm{Q}}_{{0}} ,$$ providing insight into how thermal loading affects mechanical deformation in the medium**.** When the internal heat source is present, thermal energy is continuously generated within the body, leading to enhanced thermal expansion. This thermal expansion introduces additional strain, particularly in the vertical direction, resulting in greater displacement magnitudes compared to the case without internal heating. The increase in $$v$$ due to $${\mathrm{Q}}_{{0}}$$ reflects the strong thermo-mechanical coupling, where internal temperature rise induces volumetric changes and vertical deformation, even in the absence of external mechanical forces. Figure 13 highlights the profound impact of the internal heat source $${\mathrm{Q}}_{{0}}$$ on the distribution of the thermal temperature field $$\theta ,$$ emphasizing how thermal energy generation within the medium alters heat transport and storage behavior**.** When an internal heat source is active, the system receives continuous thermal input, which results in a sustained elevation of temperature throughout the medium. This leads to higher peak values and slower thermal decay, as the added energy resists the natural tendency of the system to dissipate heat and return to thermal equilibrium. Physically, the presence of $${\mathrm{Q}}_{{0}}$$ intensifies the thermal gradients and prolongs the non-equilibrium state, effectively amplifying thermal inertia. The medium stores more thermal energy, resulting in a more pronounced and enduring thermal response. In contrast, when the internal heat source is absent $${\mathrm{(Q}}_{{0}} = 0),$$ the temperature distribution is governed only by external boundary conditions and conduction. Figure 14 presents the variation of the volume fraction field $$\psi$$ which represents micro-structural changes such as porosity, void fraction, or micro-concentration, under different thermoelastic theories and thermal loading conditions**.** The figure emphasizes how internal heat generation and the choice of theory influence the behavior of the medium’s microstructure. In the presence of an internal heat source $${\mathrm{(Q}}_{{0}} = 10),$$$$\psi$$ rises significantly, exhibiting a pronounced peak. This sharp increase results from intense thermal excitation, which drives micro-structural expansion or redistribution. The added thermal energy enhances the diffusion of mass or micro-structural constituents, leading to greater changes in volume fraction. Between the two theories, GNIII shows a slower decay of $$\psi ,$$ indicating that thermal effects persist longer in this model due to its accommodation of thermal memory and non-Fourier heat conduction. This suggests that the micro-structural response remains active for a longer duration under GNIII, making it suitable for modeling materials with sustained thermal inertia. In contrast, when no internal heat source is present $${\mathrm{(Q}}_{{0}} = 0),$$ the evolution of $$\psi$$ is more subdued. Figure 15 illustrates the evolution of the normal stress component $$\sigma_{xx}$$ as governed by the (MGT) and (GNIII) thermoelastic theories, comparing cases with and without internal heat generation. The results reveal that the internal heat source $${\mathrm{(Q}}_{{0}} = 10),$$ significantly alters the mechanical response of the medium. When the internal heat source is present, $$\sigma_{xx}$$ increases gradually and stabilizes at a relatively lower steady-state value. This behavior indicates that the thermal expansion caused by internal heating helps to partially relieve the mechanically induced stresses. Figure 16 presents the variation of the shear stress component $$\sigma_{xy}$$ under the influence of an internal heat source, emphasizing how thermal energy generation within the medium alters its mechanical response**.** In the presence of an internal heat source $${\mathrm{(Q}}_{{0}} = 10),$$ the stress magnitudes are noticeably higher and persist over a larger spatial or temporal domain. This is because internally generated heat significantly raises the local temperature, leading to stronger thermal expansion and,

consequently, greater internal strain differentials.

### Validation and limiting-case comparison

To verify the accuracy and reliability of the proposed model, a validation study was conducted by comparing the present results with previously published works obtained as special cases of the governing equations. The current formulation can be reduced to earlier thermoelastic models by neglecting selected physical effects. Specifically:

By setting the nonlocal parameter equal to zero, the model reduces to the classical local thermoelastic porous medium.

By neglecting the rotation parameter $(\Omega = 0),$ the influence of angular velocity disappears and the governing equations coincide with the non-rotating case reported in the literature.

By suppressing the internal heat source term ${\mathrm{(Q}}_{{0}} = 0),$ the formulation reduces to the corresponding thermoelastic porous medium without heat generation.

By taking the relaxation parameters associated with the Moore–Gibson–Thompson (MGT) theory to their limiting values, the governing equations recover previously established generalized thermoelastic models.

Under these limiting conditions, the numerical results for temperature, displacement, stress, and volume fraction fields were computed and compared with those reported by ^36^. Excellent agreement was observed between the present results and the published data, confirming the correctness of the mathematical formulation and numerical implementation. Figure 3 illustrates the comparison of the temperature distribution obtained from the present model and that reported in ^36^, showing nearly identical trends. Similar agreement is observed for the displacement and stress distributions presented in Figs. 2, 6. The small differences are attributable to numerical discretization and parameter selection.

These comparisons demonstrate that the proposed nonlocal thermoelastic porous model within the MGT framework accurately reproduces established results in the appropriate limiting cases and therefore provides confidence in the new results obtained when the effects of nonlocality, rotation, and internal heat source are simultaneously included.

## Conclusion

In the present work, the coupled effects of rotation, nonlocality, and internal heat generation on a porous thermoelastic medium were investigated within the framework of the Moore–Gibson–Thompson (MGT) theory and compared with the Green–Naghdi type III (GNIII) model. Based on the obtained analytical and numerical results, the following conclusions can be drawn:

- The rotation parameter has a pronounced influence on the thermoelastic response of the medium. Increasing the rotation parameter reduces the magnitude of the vertical displacement and noticeably modifies the distributions of temperature, volume fraction, and stress components due to the additional inertial effects generated by rotational motion.
- The nonlocal parameter plays a significant role in controlling the behavior of all physical fields. The inclusion of nonlocal effects smooths the spatial distributions and reduces the amplitudes of displacement, temperature, and stress fields, demonstrating the importance of small-scale interactions in porous thermoelastic materials.
- The internal heat source considerably enhances the thermal response of the medium and consequently alters the associated mechanical fields. The generated thermal energy increases the temperature field and produces noticeable changes in the displacement, volume fraction, and stress distributions through thermoelastic

coupling.

- The volume fraction field is highly sensitive to both nonlocality and internal heat generation, indicating that porosity-related effects cannot be neglected when analyzing thermoelastic wave propagation in porous materials.
- Although both MGT and GNIII theories predict similar qualitative behavior, noticeable quantitative differences are observed in all field quantities. The MGT model generally provides a more realistic description of thermal wave propagation due to the inclusion of thermal relaxation effects and finite-speed heat transport.
- The obtained results confirm that the combined action of rotation, nonlocality, and internal heat generation significantly affects the dynamic thermoelastic behavior of porous solids and should be considered in the design and analysis of advanced engineering materials operating under severe thermal and mechanical environments.

The present formulation may be useful in applications involving porous and microstructured materials, geomechanics, energy systems, aerospace structures, and other engineering problems where thermal waves, internal heat generation, and rotational effects are of practical importance.

## Data Availability

The data supporting the findings of this study were generated from analytical and numerical computations. These data are available from the corresponding author upon reasonable request.

## Nomenclature

$$\sigma_{ij}$$: Stress tensor
$$e_{ij}$$: Strain tensor
$T_{0}$: Reference temperature such that $\left| {(T - T_{0} )/T_{0} } \right| < 1$ , $\theta_{0} = T - T_{0}$
$\tau_{0}$: Relaxation time coefficient
$$K$$: Material constant
${\rm K}^{*}$: Thermal conductivity coefficient
${\boldsymbol{\varOmega}}$: Angular velocity vector
$\gamma = (3\lambda + 2\mu )\alpha_{t} ,\,\alpha_{t}$: Coefficient of linear thermal expansion
$$\delta_{ij}$$: Kronecker’s delta
$\beta ,b,\alpha_{1} ,\alpha_{2} ,\alpha_{3} ,\alpha_{4}$: The material constants due to presence of voids
$\psi$: The change in volume fraction field
$\varepsilon = a_{0} \,e_{0}$: $a_{0} \,$ An internal characteristic of length and $\,e_{0}$ constant of a material.
$\lambda ,\mu$: Lame’s constant Thermal
$$\rho$$: Density
$C_{E}$: Specific heat at constant strain
$${\mathrm{i}}$$: Imaginary number, $${\mathrm{i}} = \sqrt { - 1}$$
$$r$$: Wave number
${\mathrm{Q}}$: Moving internal heat source
${\mathrm{Q}}_{0}$: Magnitude of an internal heat source
$v_{0}$: Velocity of moving internal heat source
$f_{1} ,f_{2}$: The amplitudes of the applied thermal and mechanical loadings

## References

[1] Marin, M. Some basic theorems in elastostatics of micropolar materials with voids. *J. Comput. Appl. Math.* **70**(1), 115–126. 10.1016/0377-0427(95)00137-9 (1996).

[2] Kumar, R. & Rani, L. Deformation due to moving loads in thermoelastic body with voids. *Int. J. Appl. Mech. and Eng.* **11**(1), 37–59 (2006).

[3] Sharma, K. & Kumar, P. Propagation of plane waves and fundamental solution in thermoviscoelastic medium with voids. *J. Therm. Stress.* **36**(2), 94–111. 10.1080/01495739.2012.720545 (2013).

[4] Deswal, S. & Hooda, N. A two-dimensional problem for a rotating magneto-thermoelastic half-space with voids and gravity in a two-temperature generalized thermoelasticity theory. *J. Mech.* **31**(6), 639–651. 10.1017/jmech.2015.40 (2015).

[5] Tomar, S., Goyal, N. & Szekeres, A. Plane waves in thermo-viscoelastic material with voids under different theories of thermoelasticity. *Int. J. Appl. Mech. and Eng.* **24**(3), 691–708. 10.2478/ijame-2019-0043 (2019).

[6] Guo, Y. & Xiong, C. Influence of the viscoelastic relaxation time on a foundation under generalized poro-thermoelasticity. *Waves Random Complex Media* **34**(3), 1269–1299. 10.1080/17455030.2021.1936283 (2021).

[7] Gupta, S., Dutta, R., Das, S. & Verma, A. K. Double poro-magneto-thermo-elastic model with microtemperatures and initial stress under memory-dependent heat transfer. *J. Therm. Stress.* **46**(8), 743–774. 10.1080/01495739.2023.2202718 (2023).

[8] Othman, M. I., Said, S. M., Fathy, R. A. & Gamal, E. M. Influence of gravity and viscosity on a fiber-reinforced thermoelastic solid with voids via the three-phase-lag model. *Geomech Eng.* **39**(4), 397–405 (2024).

[9] Gupta, V., Barak, M. S. & Das, S. Impact of memory-dependent heat transfer on Rayleigh waves propagation in nonlocal piezo-thermo-elastic medium with voids. *Int. J. Numer. Meth. Heat Fluid Flow* **34**(4), 1902–1926. 10.1108/HFF-10-2023-0615 (2024).

[10] Pathania, V., Kumar, R., Gupta, V. & Barak, M. S. Generalized plane waves in a rotating thermoelastic double porous solid. *Int. J. Appl. Mech. and Eng.* **27**(4), 138–154. 10.2478/ijame-2022-0055 (2022).

[11] Pathania, V., Kumar, R., Gupta, V. & Barak, M. S. Double porous thermo- elastic waves in a homogeneous, isotropic solid with inviscid liquid. *Arch. Appl. Mech.* **93**, 1943–1962. 10.1007/s00419-023-02364-w (2023).

[12] Schoenberg, M. & Censor, D. Elastic waves in rotating media. *Quart. J. Appl. Math.* **31**, 115–125 (1973).

[13] Chand, D., Sharma, J. N. & Sud, S. P. Transient generalized magneto-thermo-elastic waves in a rotating half-space. *Int. J. Eng. Sci.* **28**(6), 547–556. 10.1016/0020-7225(90)90057-P (1990).

[14] Othman, M. I. A. & Song, Y. Q. The effect of rotation on 2-D thermal shock problems for a generalized magneto-thermoelasticity half-space under three theories. *Multi. Math. Mater. and Struct.* **5**(1), 43–58. 10.1108/15736105200900003 (2009).

[15] Singh, J. & Tomar, S. K. Plane waves in a rotating generalized thermoelastic solid with voids. *Int. J. Eng. Sci. and Technol.* **3**(2), 34–41. 10.4314/ijest.v3i2.68130 (2011).

[16] Abd-Alla, A., Abo-Dahab, S. & Khan, A. Rotational effect on thermo-elastic stoneley, Love and Rayleigh waves in fibre-reinforced anisotropic general viscoelastic media of higher order. *Struct. Eng. and Mech.* **61**(2), 221–230. 10.12989/sem.2017.61.2.221 (2017).

[17] Othman, M. I. A., Said, S. M. & Gamal, E. M. A new model of rotating non-local fiber-reinforced visco-thermoelastic solid using a modified Green-Lindsay theory. *Acta Mech.* **235**, 3167–3180. 10.1007/s00707-024-03874-6 (2024).

[18] Kalkal, K. K., Deswal, S. & Poonia, R. Two-dimensional deformations in a rotating functionally graded fiber-reinforced thermoelastic half-space with magnetic field. *Mech. Based Design of Struct.* **52**(3), 1543–1560. 10.1080/15397734.2022.2153695 (2024).

[19] Das, P. & Kanoria, M. Magneto-thermoelastic response in a functionally graded isotropic unbounded medium under a periodically varying heat source. *Int. J. Thermophys.* **30**, 2098–2121. 10.1007/s10765-009-0679-y (2009).

[20] Sarkar, N. & Lahiri, A. Interactions due to moving heat sources in generalized thermoelastic half-space using L-S model. *Int. J. Appl. Mech. and Eng.* **18**(3), 815–831. 10.2478/ijame-2013-0049 (2013).

[21] Ailawalia, P. & Singla, A. Disturbance due to internal heat source in thermoelastic solid using dual phase lag model. *Struct Eng Mech.* **56**(3), 341 (2015).

[22] Abbas, I. A. Eigenvalue approach to fractional order generalized magneto-thermoelastic medium subjected to moving heat source. *J. Magnet. and Magnet. Mater.* **377**, 452–459. 10.1016/j.jmmm.2014.10.159 (2015).

[23] Jain, K., Kalkal, K. K. & Deswal, S. Effect of heat source and gravity on a fractional order fiber reinforced thermoelastic medium. *Struct. Eng. and Mech.* **68**(2), 215–226. 10.12989/sem.2018.68.2.215 (2018).

[24] Youssef, H. M. & Al-Lehaibi, E. A. N. The boundary value problem of a three-dimensional generalized thermoelastic half-space subjected to moving rectangular heat source. *Boundary Value Problems* **2019**, Art. No.8. 10.1186/s13661-019-1119-y (2019).

[25] Zenkour, A. M., Mashat, D. S. & Allehaibi, A. M. Thermoelastic coupling response of an unbounded solid with a cylindrical cavity due to a moving heat source. *Mathematics* **10**(1), 9. 10.3390/math10010009 (2022).

[26] Das, B., Sardar, S. S., Ghosh, D. & Lahiri, A. Wave propagation in a non-local magneto-thermoelastic medium permeated by heat source. *Int. J. Comput. Methods Eng. Sci. Mech.* **24**(5), 314–327. 10.1080/15502287.2023.2186968 (2023).

[27] Said, S. M. Effect of the gravity on a nonlocal thermoelastic medium with a heat source using fractional derivative. *Geomech and Eng.* **37**(6), 591–597 (2024).

[28] Kaur, I. & Singh, K. Thermoelastic analysis of semiconducting solid sphere based on modified Moore-Gibson-Thompson heat conduction with hall Effect. *Discover Appl. Sci.* **5**, 16. 10.1007/s42452-022-05229-z (2023).

[29] Kaur, I. & Singh, K. A study of influence of Hall effect in semiconducting sphere shell with Moore-Gibson-Thompson-photo-thermoelastic model. *Iran. J. Sci. and Technol*. *Trans. Mech. Eng.* **47**, 661–671. 10.1007/s40997-022-00532-x (2023).

[30] Gupta, V., Awwad, F., Ismail, E. A. A., Ahmed, H. & Das, S. Piezo-semi- conductor-void interplay in the size-dependent dynamics of Rayleigh waves within the Moore–Gibson–Thompson framework. *Int. J. Numer. Meth. Heat Fluid Flow* **35**(9), 3400–3428. 10.1108/HFF-04-2025-0254 (2025).

[31] Das, S. et al. Hydro-thermo-electromechanical response in a size-dependent porous piezoelectric medium under memory-dependent MGT theory. *Mech Adv Mater Struct.* **10**, 1–21 (2025).

[32] Kaur, I. & Singh, K. Modified Moore-Gibson-Thompson thermoelastic model with hyperbolic two-temperatures semiconducting thermoelastic solid cylinder. *Mech. Solids* **58**, 1723–1737. 10.3103/S0025654423600745 (2023).

[33] Kaur, I. & Singh, K. Effect of nonlocal-nonsingular Fractional Moore-Gibson-Thompson theory in semiconductor cylinder. *Advances in nano research.* **15**(4), 305–313 (2023).

[34] Kaur, I. & Singh, K. Modified Moore-Gibson-Thompson thermoelastic model with two-temperatures effect on rotating semiconducting thermoelastic solid cylinder. *Int. Appl. Mech.* **60**, 370–382. 10.1007/s10778-024-01290-w (2024).

[35] Eringen, A. C. *Nonlocal Continuum Field Theories* (Springer, 2002).

[36] Othman, M. I. A., Said, S. M., Eraki, E. E. M. & Gamal, E. M. Coupled thermo-elastic effects of gravity and internal heat source in a medium with modified stress dynamics via Moore-Gibson-Thompson theory. *Results Eng.* **26**, 105401. 10.1016/j.rineng.2025.105401 (2025).

